# Potential Contributors to the Canadian Pediatric Obesity Epidemic

**DOI:** 10.5402/2011/917684

**Published:** 2011-12-05

**Authors:** Travis Saunders

**Affiliations:** ^1^Healthy Active Living and Obesity Research Group, Children's Hospital of Eastern Ontario Research Institute, Ottawa, ON, Canada K1H 8L1; ^2^School of Human Kinetics, University of Ottawa, Ottawa, ON, Canada K1N 6N5

## Abstract

As a group, Canadian children and youth are heavier than at any time in the recent past. However, to date there has been no critical examination of the factors which are likely to have contributed to these deleterious trends. A review of the evidence suggests that there is robust evidence supporting the role of reduced sleep, increased sedentary time, increased consumption of sugar-sweetened beverages, and secular increases in adult obesity as contributing factors to the current epidemic of childhood obesity. There is moderate evidence that these trends are related to changes in either total energy intake or physical activity, while there is very little evidence supporting the role of maternal age, breastfeeding, exposure to endocrine disrupters, or inadequate calcium intake. These findings suggest that targeting sleep, sedentary time, and sugar-sweetened beverage intake in Canadian children and youth may help to prevent future weight gain at the population level.

## 1. Introduction

Available evidence suggests that Canada is in the midst of an epidemic of childhood obesity [1–4]. Between 1981 and 2007–2009, the average body mass index (BMI) of 12-year-old Canadians increased from 18.1 to 19.2 kg/m^2^ in boys, and from 18.4 to 19.5 kg/m^2^ in girls [4]. During this same time period the prevalence of overweight/obesity among Canadians aged 15–19 increased dramatically from 14% to 31% in boys, and from 14% to 25% in girls [4]. In fact, among Canadians aged 15–19, fully 14% of boys and 10% of girls are now considered obese [4]. Equally worrying, as BMI has increased during the past 30 years, so too has the prevalence of abdominal obesity [1, 4]. Since 1981, the average waist circumference (WC) among Canadian youths aged 12–19 has increased by more than 5 centimetres, such that approximately one-fifth of Canadians in this age group now have a WC that places them at some form of increased health risk [1].

These recent increases in both the BMI and WC of Canadian youth are a tremendous public health concern, as pediatric obesity is associated with both metabolic dysfunction in childhood [5–8], as well as an increased risk of mortality well into adulthood [9, 10]. Thus, the objective of the present paper is to give a comprehensive examination of the possible causes of our current epidemic of childhood obesity. This will be done through a discussion of the strength of the evidence base and biological plausibility for each putative factor, before finally comparing their relative contributions to these deleterious trends. This review will focus on Canadian data whenever possible. Before examining the putative causes of the epidemic, however, it is important to briefly review the factors affecting energy balance and their role in body weight.

## 2. Energy Balance

As noted by Jéquier and Tappy, the first law of thermodynamics—which states that energy can neither be created nor destroyed—applies to humans [11]. With respect to body weight, this means that changes in stored energy (e.g., adiposity) are equal to energy intake (EI) minus energy expenditure (EE) [11]. Energy expenditure can be broken down into three separate components [11]:

(i) basal metabolic rate (BMR),

(ii) diet-induced thermogenesis, and

(iii) energy used for exercise and physical activity (PA).

Energy intake, on the other hand, is simply the sum of the energy consumed by an individual, minus approximately 5–10% that is excreted in urine and feces [11]. When EI exceeds EE, the result is an increase in energy stores, and therefore weight gain. Thus, any putative cause of the childhood obesity epidemic must influence either EI, EE, or both. With that in mind, let us now evaluate the role of both conventional and unconventional factors in the etiology of childhood obesity.

## 3. Reduced Physical Activity

Given its role in the energy balance equation it is quite obvious that, all else being equal, a reduction in the number of calories burned through PA will directly lower EE and result in positive energy balance. Regular bouts of PA are also known to result in substantial elevations in BMR in both lean and obese individuals [12, 13], suggesting that reductions in PA may further reduce EE by deleterious changes to BMR. Similarly, it has also been suggested that regular PA results in more accurate coupling of EI and EE [13–17]. Taken together, these findings suggest that reductions in PA may negatively impact both sides of the energy balance equation by directly reducing EE and by inhibiting the proper regulation of EI. Not surprisingly, available observational evidence also suggests that PA plays a role in the prevention of excess weight gain in children and youth.

Numerous cross-sectional studies report that overweight and obese children are less active than their lean peers, while the majority of longitudinal studies report small, inverse associations between high levels of PA and the accumulation of excess body weight [18–20]. For example, Berkey and colleagues report that every hour of self-reported daily PA in girls aged 9–14 is associated with a −0.0284 kg/m^2^ smaller increase in BMI over a one-year period (the relationship was of borderline significance in boys) [21]. Similarly, a recent systematic review by Connelly and colleagues reports that compulsory PA is the single most defining factor of controlled trials that successfully prevent the development of childhood overweight or obesity [22]. It should be noted that current findings are based mainly on self-reported levels of PA, which are known to be substantially less accurate than objective measures such as pedometry and accelerometry [23, 24]. However, despite these methodological limitations, the balance of evidence suggests that low levels of PA are likely to predispose to future weight gain.

While the above evidence suggests that low levels of PA are likely to result in increased risk of future weight gain, at present it is unclear whether current levels of PA in Canadian youth are lower than those of previous generations, which would be necessary in order for PA to play a causal role in the current obesity epidemic [25]. Self-reported leisure-time PA among Canadian adolescents actually increased during the 1980's and remained stable throughout the 1990's [26], suggesting that current PA levels may be higher than they were before the obesity epidemic. However, this data conflicts with other lines of evidence, which suggest that total PA levels among Canadian children may be lower than they were in the past. For example, it has been reported that the proportion of trips to school that involves active transportation decreased by roughly 20% between 1986 and 2006 among Canadian children in the Toronto region [27]. Similarly, children who live in Canadian Old Order Amish and Mennonite communities, where lifestyles are similar to those in contemporary Canadian society 60–100 years ago [23], accumulate roughly 50% more steps per day than their contemporary Canadian peers [28], as well as 30–50% more moderate-to-vigorous PA [29]. In the absence of more complete and objective data on the PA of past generations of Canadian youth and given that less than 10% of Canadian youth are currently meeting PA guidelines [30–32], it appears relatively safe to conclude that total PA-related EE of Canadian youth is at or near historic lows.

Thus, given the multiple biological mechanisms linking reduced PA with increased adiposity, consistent but relatively small longitudinal associations between PA and weight gain, and evidence suggesting that Canadian children are likely less active than in previous generations, there is currently moderate evidence that insufficient PA plays a causal role in the current epidemic of childhood obesity.

## 4. Increased Sedentary Behaviour

Sedentary behaviour is defined as “a distinct class of behaviours (e.g., sitting, watching TV, driving) characterized by little physical movement and low energy expenditure (≤1.5 METs)” [33]. At present, it is unclear whether the prevalence of sedentary behaviours in Canada and other industrialized nations has increased in recent decades. For example, a review by Marshall and colleagues reports that the screen time (time spent watching television, playing videogames, or using computers) of children in modern nations has not increased since the 1950's [34]. In contrast, Nelson and colleagues report that weekly computer usage increased by roughly 4 hours/week between 1999 and 2004 in American youth [35]. Similarly, a recent report suggests that Canadian children average more than 6 hours of screen time on weekdays, and that even preschoolers watch an average of almost 2 hours of television per day [36]—amounts that seem highly unlikely 40 years ago. Similar trends are seen in sedentary modes of transportation such as driving, which have also increased dramatically in recent decades [27]. Finally, accelerometry data from the nationally representative Canadian Health Measures Survey suggests that Canadian youth spend an average of 8.6 hours per day (more than 60% of their waking hours) engaging in sedentary behaviour [30]. Taken together, these reports suggest that Canadian children are likely more sedentary than previous generations.

In addition to reports of increasing levels of sedentary behaviour in Canadian youth, there is also an accumulating body of evidence which suggests that high levels of sedentary behaviour may predispose to weight gain, especially in young children, while reductions in sedentary behaviour may promote weight loss or weight maintenance [18, 37]. For example, Burke et al. [38] report that every hour of television watching at age 6 was associated with a 50% increased risk of overweight or obesity at age 8 in a sample of Australian children, independent of other risk factors for weight gain. Similarly, a randomized controlled trial which reduced screen time resulted in significant reductions in both weight gain and the accumulation of abdominal fat in elementary school children [39]. However, few studies have found relationships between sedentary behaviour and weight gain in older children, suggesting that sedentary behaviour may only be a risk factor for obesity in young children [18, 37].

The potential mechanisms which are thought to link sedentary behaviour and adiposity involve deleterious changes to both EE and EI. Most obviously, sedentary activities are defined by having low EE [33] and may also displace PA, although there is currently little evidence that such displacement takes place [18, 40–42]. With respect to EI, excess sedentary behaviour may also result in “uncoupling” between EE and EI [14]. Further, it has been suggested that television viewing may exert a particularly negative impact on pediatric EI. For example, Wiecha and colleagues report that every one-hour increase in daily television viewing among school children is associated with an extra consumption of 167 calories [43]. In addition, it has also been suggested that exposure to television food advertisements increases children's food intake at subsequent meals [44, 45]. Thus, through its impact on both EE and EI, it is very plausible that sedentary behaviour has a deleterious impact on energy balance, and therefore body weight.

Given the strong and consistent relationship between sedentary behaviours and weight gain in early childhood, temporal trends which suggest that sedentary behaviours have increased in recent decades, and numerous plausible biological mechanisms, there is currently strong evidence that increases in sedentary behaviour play an important role in the epidemic of childhood obesity.

## 5. Increased Total Energy Intake

It is well established that intentional overfeeding results in significant weight gain [46–48]. For example, Levine and colleagues report that overfeeding volunteers by 1000 kcal/day results in an average weight gain of 5 kg in just 8 weeks [47]. However, counterintuitively, recent reviews have noted that total EI has not been a consistent predictor of weight gain in prospective studies of children [19, 49]. It is worth noting however that this may be due to the limitations of self- or parent-reported caloric intake, as both adults and children are known to have great difficulty in accurately reporting EI [50–52]. For example, Huang and colleagues report that fully 55% of children aged 3–19 who participated in the Continuing Surveys of Food Intakes by Individuals study reported physiologically implausible values for energy intake, and that excluding these individuals resulted in dramatic improvements in both the strength and the consistency of the relationship between EI and body weight [52].

As with PA, there is currently little information regarding historical trends in the EI of Canadian children, and data from the United States are equivocal as some [53–56], but not all [57, 58], studies report increased EI during the past half century. For example, self-reported energy intake from the 1977-1978 Nationwide Food Consumption Survey and the 1999–2004 National Health and Nutrition Examination Survey suggest that the average daily EI of American children aged 1–10 in 1999–2004 was 15% higher than in 1977-1978, with similar increases observed in adolescents [54, 55]. In contrast to these findings, however, Troiano and colleagues report that between 1970 and 1994, EI in American youth was relatively stable [57]. Thus, given the absence of Canadian data and the ambiguity of available data from our closest neighbour, there is currently insufficient evidence to conclude that EI has increased in Canadian youth during recent decades.

While prospective studies and historical trends may lend only weak support to the putative role played by EI in the Canadian childhood obesity epidemic, it remains extremely plausible biologically. As mentioned earlier, several trials have shown that intentional overfeeding results in dramatic weight gain in adults [46–48], and there is little reason to expect this relationship to be different in children. While it should be noted that there is evidence that over-consumption results in compensatory increases in EE in some individuals, [47], this would likely be insufficient to prevent an increase in obesity rates at the population level. Further, other consistent predictors of weight gain which lend themselves to more accurate self-reporting than total EI (e.g., television watching and sugar-sweetened beverages, which will be discussed below) are thought to exert their influence through their impact on EI. Thus, despite the weak evidence presented from observational studies, the strong biological plausibility and impressive results from studies of chronic overfeeding suggest that there is currently moderate evidence that increased EI has contributed to the childhood obesity epidemic.

## 6. Increased Sugar-Sweetened Beverage Intake

While trends in total EI over the past 40 years are unclear, there is little ambiguity for trends in sugar-sweetened beverage (SSB) intake, which has increased dramatically in recent decades [58–60]. For example, the average self-reported soft-drink intake in American youth increased from roughly 150 mL/day in 1977 to more than 350 mL/day in 1998 [59], and recent studies suggest that total SSB intake has continued to increase into the 21st century [60]. Interestingly, while this may be partially due to increased fast food consumption, available evidence suggests that SSB intake has also increased in the home environment in recent decades [56].

Several recent systematic reviews have also concluded that there is consistent evidence that excess consumption of SSBs is associated with an elevated risk of weight gain [19, 61, 62]. For example, among longitudinal studies, Vartanian and colleagues report significant effect sizes of 0.24 and 0.09 for the relationship of SSB consumption with total EI and body weight, respectively [62]. Similarly, a 19-month prospective study of 548 school children reports that every serving of sugar-sweetened beverages at baseline was associated with a 0.18 kg/m^2^ increase in BMI at followup [63]. Finally, it has recently been estimated that removing sugar-sweetened beverages from the diet of American children and youth would reduce caloric intake by an average of 235 calories per day [64], which has the potential to dramatically reduce the risk of positive energy balance in this age group. Thus, available evidence suggests that excessive consumption of SSBs plays a strong role in the etiology of the childhood obesity epidemic.

This relationship between SSB and prospective weight gain can be explained by multiple biological mechanisms. First and foremost, SSB intake is associated with increased EI, as described earlier [62]. This is likely due to the fact that SSBs are both energy dense and have little impact on satiety, both of which could lead to increases in EI [61]. Further, many SSBs are sweetened with high fructose corn syrup (HFCS) and therefore contain a fructose fraction. This fructose fraction may also contribute to weight gain through increased lipogenesis, inhibition of satiety signals, and reductions in EE, although it should be noted that the relative importance of HFCS in the etiology of obesity is still a matter of dispute [61, 65].

## 7. Increased Dietary Fat Intake

Not surprisingly, as the consumption of carbohydrates has increased during the past 30 years, the relative contribution of fat to total EI has decreased, although intake remains above recommendations [57, 58]. For example, Cavadini and colleagues report that between 1965 and 1996 fat intake decreased from 39% to 32% of EI for Americans aged 11–18 [58]. However, it should be noted that this same study found that *absolute *fat intake actually increased by 4% during the 1990's [58], suggesting that the relative changes in fat intake may have more to do with increased consumption of carbohydrates than with reductions in fat consumption.

The evidence linking fat intake and obesity in prospective studies is surprisingly equivocal and provides little support for the role of fat intake in the development of obesity [19]. For example, Davis and colleagues report that of 15 longitudinal studies of childhood weight gain reviewed by the American Dietetic Association, just 4 supported the role of dietary fat intake, while 4 others showed mixed results, and 7 found no association [19]. However, as with total EI, fat intake remains an extremely plausible mechanism biologically. Similar to SSBs, dietary fat is both energy dense and relatively nonsatiating per calorie ingested, lending itself to passive overconsumption [66]. Further, increased fat consumption appears to have little impact on fat oxidation or overall EE, suggesting that excess EI related to increased fat intake is very likely to result in positive energy balance and weight gain [67]. However, despite these plausible mechanistic links, available evidence provides only weak support of the role of dietary fat intake in the current childhood obesity epidemic.

## 8. Reduced Calcium Intake

Along with the changes in fat and SSB intake in recent decades, there has also been a well-documented reduction in the intake of dietary calcium [58, 68]. For example, between 1965 and 1996, Cavadini et al. report that the milk consumption of American youth decreased by 36%, while total calcium intake dropped by roughly 13% [58]. Further, it has been suggested that high calcium intake may influence body weight through increases in fecal fat excretion, fat oxidation, and thermogenesis [69, 70]. However, a recent meta-analysis of childhood calcium supplementation studies reports no significant association between supplementation and any measure of weight or body composition [71], which is supported by the findings of a similar review in adults [72]. Thus, while there is some evidence for plausible mechanisms linking reduced calcium with increased adiposity, the lack of evidence linking calcium intake with changes in actual measures of body composition suggests that reductions in calcium intake do not represent an important cause of the Canadian childhood obesity epidemic.

## 9. Reduced Sleep

Available evidence suggests that short-sleep duration may be another important risk factor for childhood overweight and obesity. A recent systematic review and meta-analysis by Cappuccio and colleagues reports that children who sleep less than 10 hours per night are at 89% greater risk than their peers who sleep more than 10 hours/night [73]. Using this same data, it has been estimated that 5 to 13% of all childhood obesity could be due to short-sleep duration [74]. Although the vast majority of the research to date has been cross-sectional [73], there is evidence of sleep as a predictor of weight gain in prospective studies as well. For example, Reilly and colleagues report that toddlers who slept less than 11 hours per night at age 2.5 years were 35–45% more likely to be obese at age 7 than toddlers who averaged more than 12 hours of sleep [75], with similar findings reported by Bell and Zimmerman [76].

Secular trends in sleep duration also support the putative role of sleep duration in the childhood obesity epidemic. Since the 1970's, the average sleep duration of children has decreased significantly among industrialized nations. Between 1974 and 1986, the average sleep time of 2-year olds in the Zurich Longitudinal Studies decreased by 45 minutes [77], while Dollman and colleagues report a 30-minute decrease from 1985 to 2004 among South Australian teenagers [78]. Similarly, the prevalence of sleep-onset difficulties has also increased dramatically in recent years [79].

Finally, a putative role for shortened sleep in the etiology of the obesity epidemic is also supported by plausible mechanisms which are thought to influence both EE and EI [80, 81]. For example, it has been reported that sleep restriction in adults results in significant increases in hormones which promote EI including cortisol and ghrelin, along with decreases in anorectic hormones such as leptin and PYY [80, 82–84]. Not surprisingly, short-sleep duration has also been shown to result in increased hunger and appetite, both of which were strongly associated with the changes in ghrelin and leptin mentioned earlier [84]. Given that leptin and ghrelin are thought to, respectively, promote and inhibit physical activity, it has been suggested that sleep debt could potentially result in reductions in EE as well [81, 85]. However, recent experimental evidence in young men suggests that acute sleep restriction results in relatively little change in EE [86]. Thus, at present it appears very likely that sleep deprivation results in increased EI, while there is little direct evidence that it results in reduced EE. When these biological mechanisms are considered alongside the consistent relationship between shortened sleep and obesity in prospective studies, and secular trends in sleep duration, there is currently strong evidence that shortened sleep plays a role in the childhood obesity epidemic.

## 10. Prenatal Exposure to Endocrine-Disrupting Chemicals

Endocrine-disrupting chemicals (EDCs) are any “compound, either natural or synthetic, which alters the hormonal and homeostatic systems that enable the organism to communicate with and respond to its environment” [87], several of which (known as obesogens) may influence body weight [88]. Limited evidence suggests that EDCs may exert a negative influence on aspects of EE. For example, it has been reported that mothers who have high levels of polychlorinated biphenyls (PCBs) in their breast milk also have low levels of plasma triiodothyronine, a thyroid hormone which is known to stimulate basal metabolism [89]. Similarly, interventions in adults which increase plasma organochlorine concentrations result in significant decreases in both triiodothyronine and resting metabolic rate [90] and may also reduce skeletal muscle oxidative capacity [91]. However, despite this limited biological evidence linking EDCs and EE, at present it is unclear whether prenatal exposure to EDCs predisposes to future weight gain [92]. For example, while some reports suggest that the concentration of PCBs in cord blood is positively associated with BMI in early childhood [92], other reports suggest no relationship [93], or even a negative relationship [94] between prenatal PCB exposure and prospective weight gain. Similar inconsistencies have also been observed for other EDCs such as DDE [92]. Thus, while being an interesting area for future research, at present there is very little evidence that EDCs play a causal role in the childhood obesity epidemic.

## 11. Increased Maternal Age

The average age of first pregnancy has increased dramatically in recent decades in both Canada [95–97] and around the world [98–100], and several plausible mechanisms have been suggested, which could link maternal age with increased risk of childhood obesity. For example, older mothers are known to give birth to smaller infants, which is itself a risk factor for the development of obesity [96, 101]. Similarly, older women are also likely to have both higher plasma concentrations of EDCs and higher BMIs, both of which may also predispose their children to future weight gain, as discussed elsewhere in this review [102–104]. Finally, research in sheep suggests that older maternal age may result in increased fat deposition [105], which may be related to accelerated reductions of proteins responsible for thermogenesis-related energy expenditure [25], although it is not immediately clear how or if this relates to humans.

Although the mechanisms described above are all at least somewhat plausible, the relationship between maternal age and childhood obesity in observational studies is inconsistent. For example, while Patterson and colleagues report that the odds of obesity in a cohort of American girls increased by 14% for every 5-year increase in maternal age [106], a more recent study of 8234 British children found no relationship between maternal age and risk of obesity at age 7 [75]. Given this conflicting evidence, there is currently only weak evidence that maternal age plays a role in the childhood obesity epidemic, and future prospective studies are needed to clarify this relationship.

## 12. Reduced Breastfeeding

Duration of breastfeeding has been strongly and consistently linked with reduced risk of childhood overweight and obesity [107]. For example, Harder and colleagues performed a meta-analysis which examined the association between duration of breastfeeding and the risk of childhood overweight in 17 independent observational studies [107]. In comparison to children who were breastfed for less than 1 month, they report that children who were breastfed for 1–3 months had 19% reduced risk of overweight. The risk of being overweight continued to decrease as the duration of breastfeeding increased—risk was reduced by 24% among those breastfed for 4–6 months, 33% among those breastfed for 7–9 months, and by 50% for those breastfed for more than 9 months. On average, each additional month of breastfeeding reduced the risk of being overweight by 4%.

Despite consistent reports of the relationship between breast feeding and reduced risk of overweight and obesity, the mechanisms underpinning this relationship remain unclear. It has been suggested that it may be due to alterations in the neuroendocrine control of appetite, although this has yet to be verified in human participants [107]. Thus, it is not possible at present to determine the precise mechanisms linking the duration of breastfeeding to body weight in childhood. 

While breastfeeding appears to have a strong relationship with the risk of excess weight gain in childhood, trends in the prevalence of breastfeeding suggest that it is not a major contributor to secular increases in childhood obesity rates during the 20th century. Since the 1970's, the prevalence of breastfeeding has remained constant or increased among most western nations for which data is available [108, 109]. For example, in the early 1970's roughly 20% of American women exclusively breastfed while in the hospital, but this increased to 45% by the year 2000 [109]. Given that obesity rates continued to increase steadily throughout this period despite increases in the prevalence of breastfeeding, there is currently weak evidence that breastfeeding plays a primary role in the childhood obesity epidemic.

## 13. Increased Adult Obesity Rates

Available evidence suggests that both parental obesity and gestational weight gain are risk factors for childhood obesity [75, 110, 111]. For example, Reilly and colleagues examined the relationship between parental and childhood obesity in a prospective study of nearly 9,000 British children [75]. In comparison to children born to two nonobese parents, they report that children were 2.5 times more likely to be obese when they had an obese father, and 4.3 more likely to be obese if they had an obese mother. Further, children born to two obese parents were more than 10 times more likely to develop obesity by age 7 than those born to two non-obese parents [75]. It has been reported that gestational weight gain is also a predictor of childhood obesity, and that this impact is stronger in women who were obese prior to becoming pregnant [111]. Finally, recent reports suggest that surgical weight loss prior to pregnancy dramatically reduces the risk of childhood obesity in babies born to obese women [112]. These relationships suggest that any putative cause of the increasing prevalence of adult obesity [113] including those that are unlikely to play a direct role in the epidemic of childhood obesity (e.g., iatrogenic weight gain [25]) may nonetheless play important indirect roles.

The relationship between parental and childhood obesity is likely to be linked via numerous mechanisms. For example, genetic factors are reported to account for roughly 25% of the variance in fat mass [114], which is likely to mediate some of the relationship in body composition between parent and child. Further, learned behavioural characteristics such as food choices, PA, and sedentary behaviours are also likely to mediate the transmission of intergenerational obesity [75, 115]. Finally, studies of animal models suggest that obesity or excessive weight gain during pregnancy is likely to predispose childhood obesity through deleterious changes in the central regulation of energy balance [116]. For example, lambs born to overfed ewes are less sensitive to signals of excess nutrient supply or fat mass than lambs born to control animals [116]. Taken together, the strong association between parental and childhood obesity and the numerous plausible mechanisms underlying these associations suggest that one of the most important drivers of the childhood obesity epidemic may in fact be adult obesity.

## 14. Relative Contributions to Childhood Obesity

As has been noted by others, there is currently insufficient information to make a truly objective ranking of the putative causes of an issue as complex as the current obesity epidemic [25]. However, the evidence presented above does allow some general conclusions to be made. This review has identified 4 factors—reduced sleep, increased sedentary time, increased consumption of sugar-sweetened beverages, and secular increases in adult obesity—which are likely to have made an important contribution to Canada's childhood obesity epidemic. Each of these factors has shown strong and consistent associations with childhood weight gain, has increased in prevalence during the obesity epidemic, and results in either biological or behavioural changes that are likely to promote positive energy balance. Of these, adult obesity appears to have the most powerful impact on childhood obesity levels, while reducing the consumption of sugar-sweetened beverages may be among the simplest ways to prevent future weight gain in individuals of all ages.

Available evidence provides only moderate support for the role of either total EI or PA in the etiology of childhood obesity. This is likely due to methodological limitations of self-reported intake and expenditure, as both of these factors are biologically plausible and have been shown to have impressive effects on adiposity in experimental studies. It is possible that methodological limitations may also explain the inconsistent relationships seen between obesity and dietary fat intake. Future studies employing more objective methods of measurement are important to determine the true role of these factors in the etiology of the childhood obesity epidemic.

Finally, although each has been linked in some way with childhood obesity, there is currently weak evidence supporting the role of maternal age, breastfeeding, exposure to endocrine disrupters, or calcium insufficiency in the etiology of the childhood obesity epidemic. Of these, maternal age, breastfeeding, and endocrine disruptors appear worthy of future study, while there is sufficient evidence to conclude that calcium intake plays little role in pediatric obesity rates at the population level.

## 15. Summary

Although influenced by numerous factors, available evidence suggests that the Canadian childhood obesity epidemic is most closely related to deleterious changes in sugar-sweetened beverage intake, sedentary behaviour, reduced sleep, and adult obesity. Interventions aimed at modifying these factors may help to prevent further increases in obesity rates among the Canadian pediatric population.
